# Possible species-flock scenario for the evolution of the cyprinid genus *Capoeta* (Cypriniformes: Cyprinidae) within late Neogene lake systems of the Armenian Highland

**DOI:** 10.1371/journal.pone.0215543

**Published:** 2019-05-08

**Authors:** Anna Ayvazyan, Davit Vasilyan, Madelaine Böhme

**Affiliations:** 1 Department of Geosciences, Eberhard-Karls-University Tübingen, Tübingen, Germany; 2 Senckenberg Center for Human Evolution and Palaeoenvironment (HEP), Tübingen, Germany; 3 JURASSICA Museum, Porrentruy, Switzerland; 4 Department of Geosciences, University of Fribourg, Fribourg, Switzerland; University of Vienna, AUSTRIA

## Abstract

We studied 4 Ma old isolated pharyngeal teeth from lake sediments of Çevirme (Tekman Palaeolake, Erzurum Province). Based on shape characters defined for 3D models of modern species, we found that the Pliocene lake constitutes sympatric occurrence of four *Capoeta* species (*C*. cf. *umbla*, *C*. cf. *baliki*, *C*. cf. *sieboldi* and *C*. sp. *sevangi/capoeta*), whose modern relatives belong to a monophyletic clade inhabiting today three different drainage systems of this region (Euphrates River, Kura River and Black Sea). We interpreted this high local diversity of closely related species in terms of the species-flock model. The Tekman palaeolake was a part of an unrecognized extended late Miocene to Pliocene palaeolake system in the present-day Armenian Highland, which has been disrupted by Pliocene tectonic activities. Surface uplift of the Armenian Highland contributed to the very characteristic biogeographic distribution and endemism of *Capoeta* in West Asian drainage systems. Thus, we proposed a species-flock scenario for the evolution and dispersal of the cyprinid genus *Capoeta* in a huge unrecognized palaeolake system in the present-day Armenian Highland.

## Introduction

Tigris and Euphrates are the largest rivers in Western Asia, both have a rich and diverse aquatic fauna which includes seven endemic fish genera (two of the Cobitidae family and five of the Cyprinidae family) [1]. In southern Caucasus, the Kura-Araxes River Basin is the major river system with many tributaries [2]. It is also characterized by several endemic fish species [3]. Among them, the cyprinid genus *Capoeta* shows phylobiogeographical pattern and it is widely distributed in Western Asia and the Ponto-Caspian region (Euphrates, Tigris, Araxes, Kura and Orontes) with 30 valid species [4, 5]. The distribution of the *Capoeta* species within Western Asian and Ponto-Caspian water basins provides an excellent basis for the analyses biogeographical evolution of the main drainage systems of this region.

Western Asia situated on the border of three continents (Europe, Asia, Africa), plays an important role in the dispersal of different groups of organisms. The area, having complex topography, provides various habitats and creates unique ecosystems. The territory is formed during a long and complex geological history, driven by the tectonic convergence of the Afro-Arabian and Eurasian plates [6]. Despite its biogeographic bridging position between the Palearctic, Afrotropic and Indomalaya realms, its deep-time biodiversity has not been studied, hampering comprehensive interpretation of the present diversity and its potential role in evolutionary processes. Western Asian and Ponto-Caspian drainage system include rather short and small but numerous drainage systems of the Mediterranean Sea Basin (this territory includes southern Anatolia, Syria, Lebanon, Israel, the Arabian Peninsula), Black Sea Basin (northern Anatolia and Western Georgia), the Tigris-Euphrates (Persian Sea Basin) and Kura-Araxes basins, most of Iranian territory (Caspian Sea Basin) [1].

**Fig 1:**
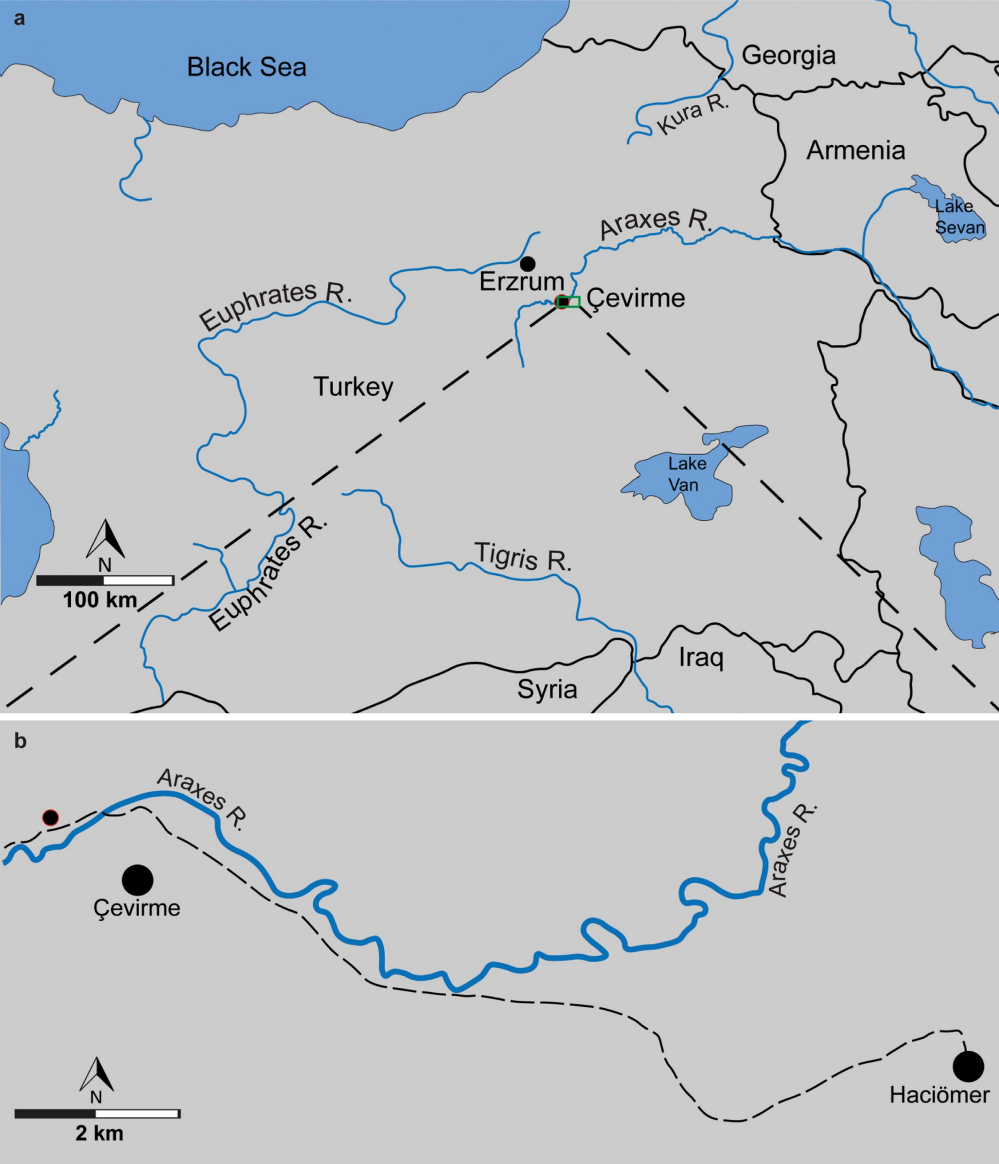
The Armenian Highland. (**a**), Fossil locality marked by red contoured circle in a relation to the Euphrates-Tigris and Araxes-Kura water basins. (**b**), map showing the fossil locality marked by red contoured circle. Map data: Fig 1A and 1B is redrawn and modified from U. S. Geological Survey, CC BY 4.0.

The four main rivers of Western Asia and the Ponto-Caspian region (Euphrates, Tigris, Kura and Araxes) all originate in the Armenian Highland (Fig 1A). The history and formation of these water basins remain largely unknown. To track the evolution of drainage basins, fossil records of aquatic faunas can be used. Recently, Vasilyan & Carnevale (2013) shown, using the fossil record of the genus *Garra* from Armenia, that area including the upper reaches of the present-day Araxes River drainage system belonged to the Protoeuphrates-Tigris drainage system in the latest Miocene [7, 8] [earlier [7] the age of the locality has been dated to Pliocene, the new results [8] suggest slightly older age latest Miocene].

In the present study, we trace back the fossil record of the genus *Capoeta* to 4 Ma, using fossil material found at the Pliocene age locality Çevirme (Erzurum Province, Tekman district) in Eastern Turkey (Fig 1A and 1B). The study sets the following goals: (1) to apply the established methodology [9] for species-level identification of isolated pharyngeal teeth of *Capoeta*; (2) to determine species composition within the fossil sample; (3) to evaluate the history and coverage of lacustrine sediments in Western Asia and the Ponto-Caspian region; and (4) to discuss evolutionary models for the genus *Capoeta* with respect to its biogeography.

### Species flock concept in ichthyology

A species flock is a monophyletic group of closely related sympatric species inhabiting the same area or geographically restricted area. The species flock is common for both vertebrate and invertebrate animals, which show rapid adaptive radiation, morphological divergence and speciation [10–13]. The examples of species flock are recorded in different groups of animals: insects, fishes, lizards and birds, [14–19]. Especially monophyletic groups of fishes represent a particular interest, as one of the criteria of the species to be considered as a species flock is the monophyly of the described groups/species [20, 21].

Two main well-known species flocks of cyprinids fishes are found in the Philippine Lake Lanao and the Ethiopian Lake Tana [18, 22–24]. Besides the extant species flocks, some potential fossil species flocks are also reported, e.g., from the Eocene site in Tanzania, the upper Miocene Lukeino Formation in the Tugen Hills of the Central Rift Valley of Kenya [17].

### Cyprinid pharyngeal dentition

The oral jaws (e.g. dentary, maxilla, premaxilla) of cyprinids are toothless. Instead they have pharyngeal teeth located on the pharyngeal bones [25]. Both left and right fifth ceratobranchials are modified into pharyngeal jaws, which have the function of food processing [25, 26]. The pharyngeal bones and teeth provide important taxonomic characters for systematics of the cyprinid fishes. The number and arrangement of the pharyngeal teeth in tooth rows are recognized and widely used diagnostic characters for cyprinid classification [27].

The fossil remains of cyprinids are mainly represented by isolated pharyngeal teeth [28] and it is hard to identify specimens based on sole isolated teeth. Therefore, the fossil record of many cyprinids, included the genus *Capoeta*, is still largely unknown.

### The fossil record of the genus *Capoeta*

According to the molecular data, the genus *Capoeta* originates around the Langhian–Serravallian boundary (13.9 Ma) and diversification within the genus occurs along the Middle Miocene–Late Pliocene period [29].

**Fig 2:**
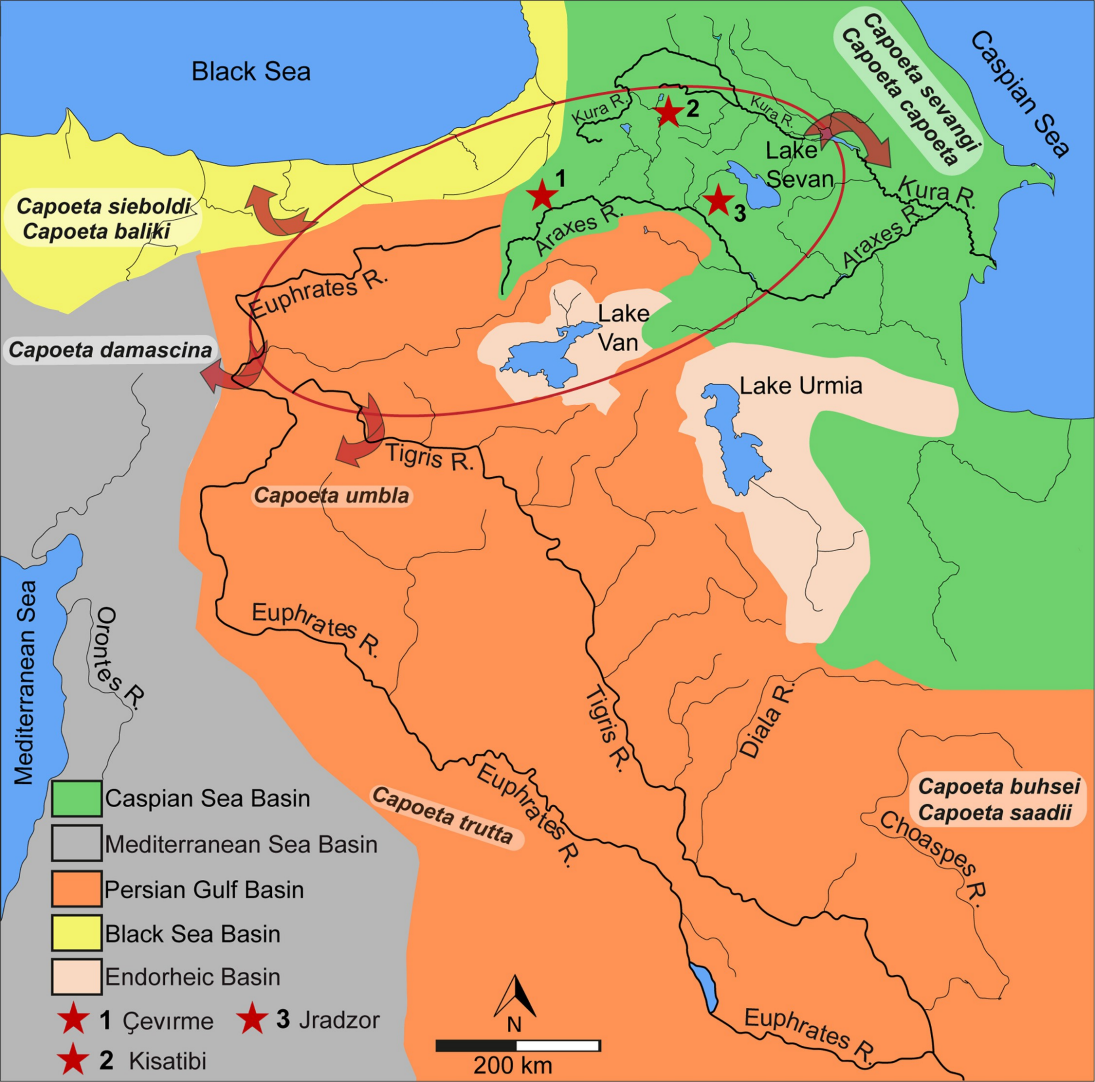
Geographical overview of the drainage systems of Western Asia and the Ponto-Caspian regions (Euphrates-Tigris, Araxes-Kura). Red star (1) indicates the position of the Ҫevırme locality. The red circle shows the possible extension of palaeolake system of the Armenian Highland. The arrows show the late distribution of the recorded fossil *Capoeta* species into the different water basins due to the tectonic disruption of the Lake system during the Pliocene uplift period. The two already known late Miocene fossil sites Kisatibi (red star 2) and Jradzor (red star 3) are included as well. Map data: Fig 2 is redrawn and modified from U. S. Geological Survey CC BY 4.0.

The scarce fossil record of *Capoeta* comes from four localities, two from the late Miocene and two from the Pleistocene. Miocene *Capoeta* fossils are known from Armenia and Georgia, both in the present-day Kura-Araxes drainage basin (Fig 2). The first fossil remains of *Capoeta nuntiu*s are described by Bogachev (1927) at the late Miocene locality in the Kisatibi, Samtskhe-Javakheti region, Georgia [30, 31]. The material is represented by three more or less complete and a few strongly damaged skeletons as well as more than 70 isolated bone fragments. Vasilyan & Carnevale (2013) describe skeletons of *Capoeta* sp. from the Jradzor locality (latest Miocene) in Armenia [32]. The record of *Capoeta* from late Pliocene sediments at Ericek (Cameli Basin, SW Anatolia) is doubtful [33], since the tooth morphologies (Fig 4A–4D in [33]) are not found within pharyngeal teeth of the *Capoeta* species. Instead of this, they resemble the morphology of the genus *Luciobarbus*; as the reported cobitid and gobiid remains are snake jawbones. Vasilyan et al. (2014) describe two isolated pharyngeal teeth and two fragments of serrated dorsal fin rays referred to *Capoeta* sp. from the early Pleistocene locality Pasinler (Erzurum Province, north-eastern Turkey). Fossil remains of *Capoeta damascina* Valenciennes, 1842 are also recorded from the Hula Palaeolake [34]. The site is situated in the northern part of the Dead Sea Rift, Israel and dated to the Middle Pleistocene (0.78 Ma).

### Biogeographical distribution of extant *Capoeta* species

**Fig 3:**
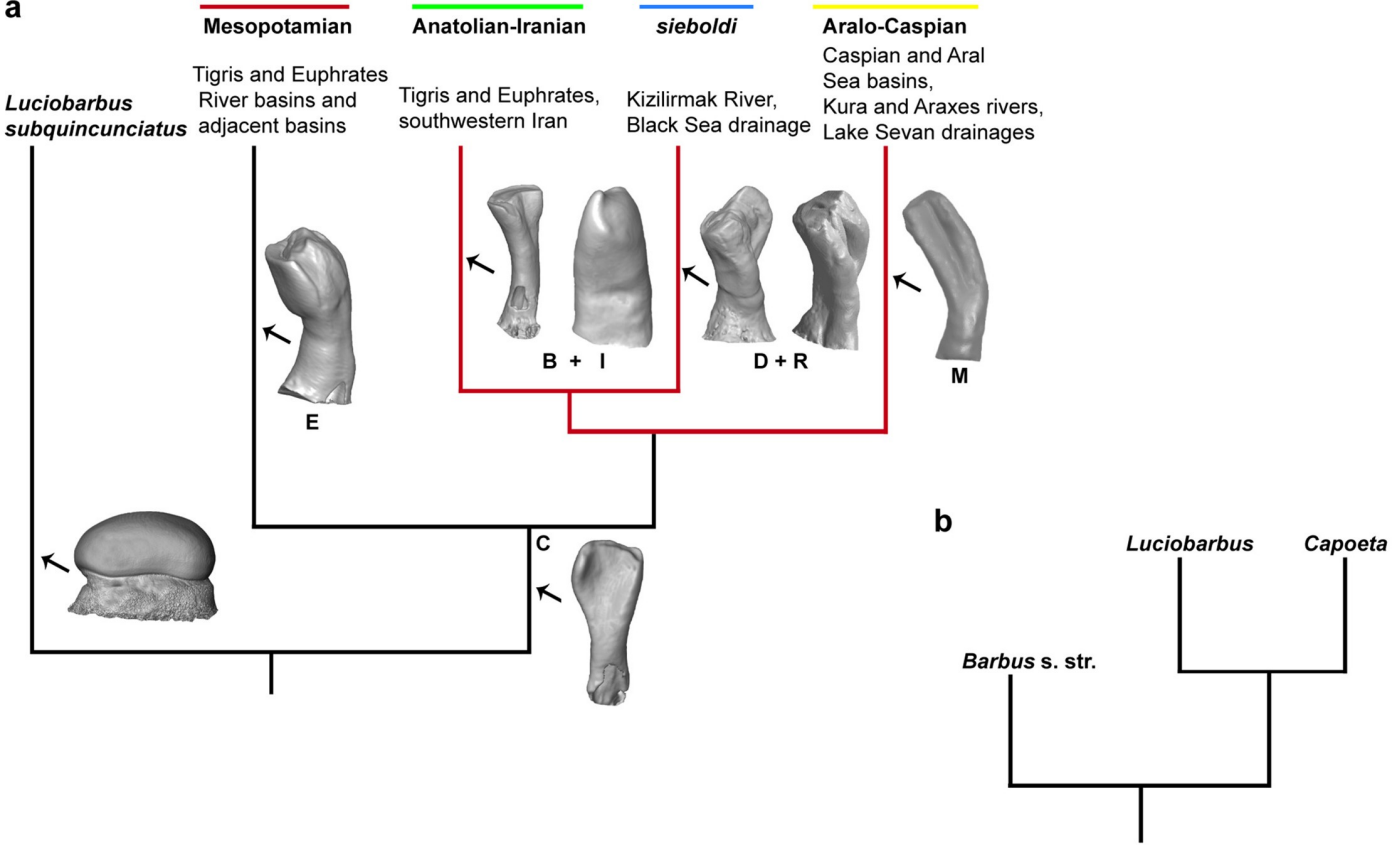
Phylogeny of the genus *Capoeta*. (**a**), distinguished clades within the genus *Capoeta* (*Luciobarbus suquincunciatus* is the sister clade) (Levin et al., 2012). The clade diagnostic shape classes of recorded clades within the fossil material (see, Ayvazyan et al. 2018; Fig 7) are given in capital letters included 3D images of teeth of *Capoeta* as well as the a2 tooth of *L*. *subquincunciatus*. The monophyletic Anatolia-Iranian/Aralo-Caspian/*sieboldi* clade, for which we propose a species flock model of evolution, is marked by red colour. (**b**), the location of *Capoeta* clade within phylogenetic tree based on the molecular genetic analysis (Levin et al., 2012).

According to the molecular data, the monophyletic genus *Capoeta* is represented by three main clades: Mesopotamian, Anatolian-Iranian and Aralo-Caspian clades and nested within the genus *Luciobarbus* as a sister group of the species *Luciobarbus subquincunciatus* [29, 35, 36] (Fig 3A and 3B). The Mesopotamian group contains species distributed in the Tigris-Euphrates drainage system and adjacent water basins: *Capoeta trutta* (Heckel, 1843), *Capoeta turani* Özulu & Freyhof, 2008 and *Capoeta barroisi* Lortet, 1894. The Anatolian-Iranian group includes species inhabiting the Black Sea Basin: *Capoeta sieboldi* Steindachner, 1864, *Capoeta baliki* Turan, Kottelat, Ekmekçi & Imamoglu, 2006, *Capoeta banarescui* Turan, Kottelat, Ekmekçi & Imamoglu, 2006. The Mediterranean drainage basins (Anatolian-Iranian clade) of southeastern Turkey, the Tigris–Euphrates river system, and small rivers, which drain into the gulfs of Persia and Oman, as well as inland water bodies in Iran contain the following species: *Capoeta buhsei* Kessler, 1877, *Capoeta saadii* (Heckel, 1847), *Capoeta caelestis* Schöter, Özulu & Freyhof, 2009, *Capoeta damascina*, *Capoeta angorae* (Hankό, 1925) and *Capoeta kosswigi* Karaman, 1969. Finally, the Aralo-Caspian group includes the species distributed in the Kura and Araxes rivers, as well as Aral and Caspian Sea drainages: *Capoeta capoeta* Güldenstädt, 1773, *Capoeta sevangi* De Filippi, 1865, *Capoeta aculeata* (Valenciennes, 1844) (S1 Table) [29].

A recent phylogenetic analysis [9], using the morphologies of pharyngeal teeth of ten *Capoeta* species, groups them in four main clades. Three of these clades show the same tree topography that the molecular data provides, the remaining clade groups differently [9].

### Late Neogene lacustrine sedimentation in the Armenian Highland

Present-day Armenian Highland (Eastern Anatolia, Armenia, Iranian Azerbaijan, Samtskhe-Javakheti region of Georgia) is composed of the high mountainous landscapes of the Eastern Taurides with elevations between 1.700 to over 5.000 meters above sea level. Because of the dominant arid climate during the late Holocene, lakes are rare in this region. Two endorheic saline lakes, Lake Van and Lake Urmia, as well as the freshwater Lake Sevan are notable exceptions (Figs 1A and 2). However, geologic mapping revealed, that during the pre-Quaternary lacustrine, sedimentation was widespread and long lasting in this region. According to Altınlı (1966) during the Late Miocene and Pliocene (11.6–2.6 Ma) lacustrine sedimentation dominated Eastern Anatolia with regional thicknesses of deposits over 1.000 m. These sediments contain a rich freshwater fauna (e.g. diatoms, gastropods, bivalvs, ostracods, fishes); [37–42] and have been variously attributed to the Horasan Formation, Gelinkaya Formation, Işıklar Formation (all in the Erzurum Province), Zırnak Formation (Bitlis Province), Ҫaybaği Formation (Elazığ Province), or to the Parҫikan Formation (Malatya Province). Despite extensive syn-sedimentary volcanism, none of these formations are radiometrically dated, but available K-Ar data [43] and rare rodent fossils [44, 45] suggest that the main lacustrine phase in Eastern Anatolia centred between 6 and 3 Ma, probably coeval with the supposed uplift of this region [46].

An older lacustrine period is documented in Iranian Azerbaijan, where fish bearing (Atherinidae, Cyprinodontidae, Leuciscinae, but no Barbinae) lake sediments from the Tabriz Basin (‘lignite beds’, ‘fish beds’) have been dated to between 12 and 7.5 Ma [47].

These late Neogene lacustrine sediments have tectonically fragmented exposure over a huge area in the Eastern Taurides stretching several hundreds of kilometres, notably including the upper reaches of present-day Euphrates, Tigris, Kura and Araxes rivers (Fig 2).

### Fossil locality Ҫevırme

The fossil site Ҫevırme (Erzurum Province, Tekman district) is located 12 km west of the Haciömer village on the road from Haciömer to Tekman, 500 m after the bridge over the Araxes River (coordinates: N 39° 37´ 37°´; E 41° 38´; Figs 1A, 1B and 2). The locality belongs to the Tekman Basin (East-Anatolian Taurides), approximately 40 km south from the Pasinler Basin and 120 km north-northwest of Lake Van. Late Neogene sediments in the Tekman Basin laying discordant over early Miocene marine limestones [48]. The sedimentary facies of the basin infill change from fluvial-alluvial to lacustrine. The late Miocene sedimentary formation (Hacıömer Formation) is composed of an approximately 300 m thick reddish-brown sequence of conglomerates, sandstone and silts with minor intercalation of marls. In the south of the basin, the alteration with vulcanites appear. These terrestrial-fluvial fossil free layers intercalate in their upper parts with nearly 200 m thick lacustrine sediments of the Işıklar Formation, which mainly consist of light grey, as well as slightly reddish freshwater carbonates (Fig 1B). Layers of marl, organic rich clay and tufa are also present. The section is covered by Pleistocene basalts from the Bingöl Dag area [48].

**Fig 4:**
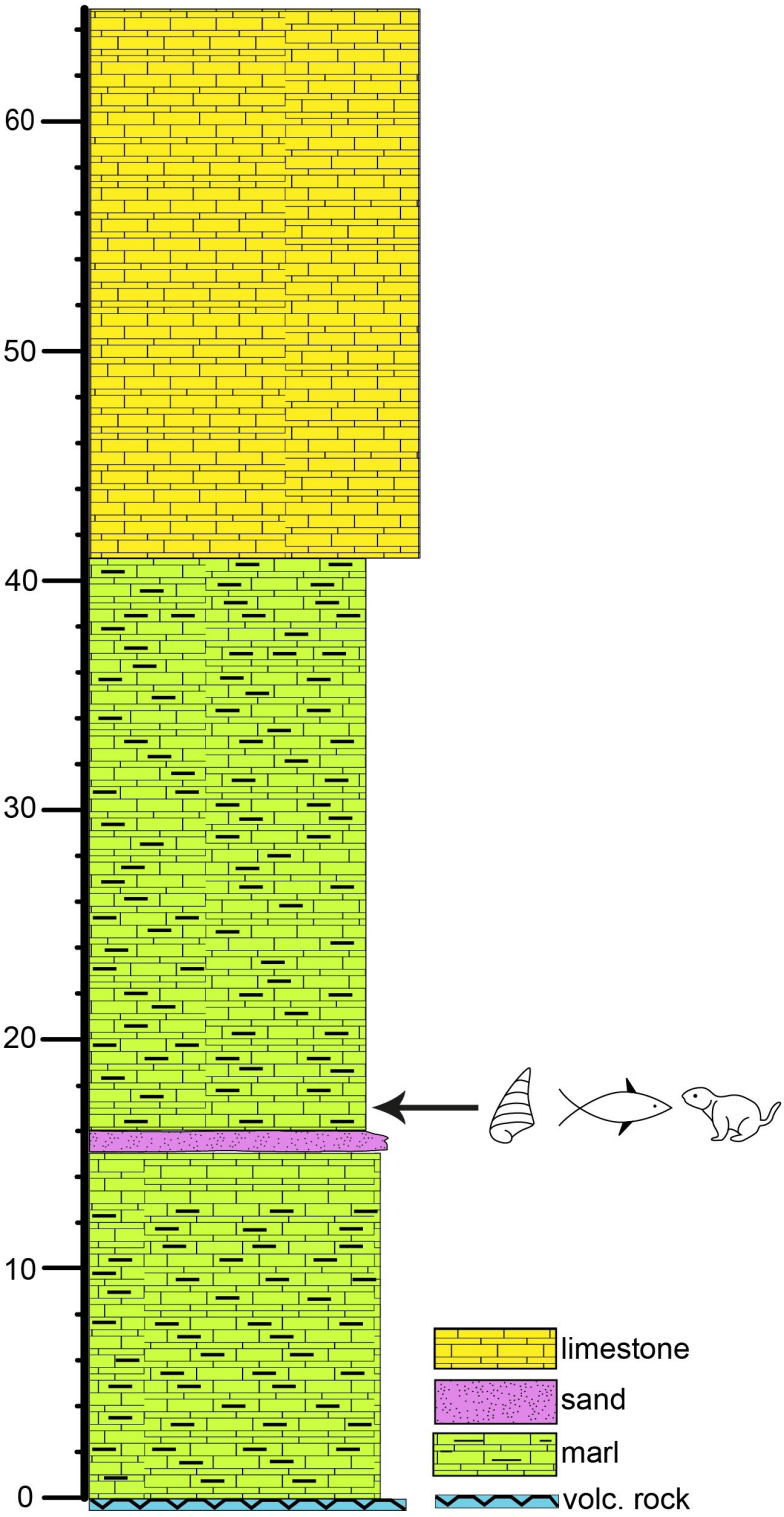
Sedimentary succession of the Işιklar Formation at the fossil site. Çevirme (Erzurum Province, Tekman district) according to Sickenberg (1975).

The fossil site Ҫevırme, discovered and first described by Sickenberg (1975), belongs to the lacustrine upper part of the Işıklar Formation. The 65 m thick stratigraphic section is subdivided based on lithological and sedimentological characters. The fossil remains of fishes, molluscs and mammals are found at 18 m depth of the section (Fig 4). Earlier palynological studies at Ҫevırme section indicate an early Pliocene pollen spectrum, which is in accordance to the small mammal fauna [48, 49]. A recent preliminary taxonomic update of the rodent association reveals, among others, the genera *Mimomys* and *Occitanomys* and a MN15a biostratigraphic position, roughly of about 4 Ma in the middle part of the Pliocene [44].

## Material and methods

**Fig 5:**
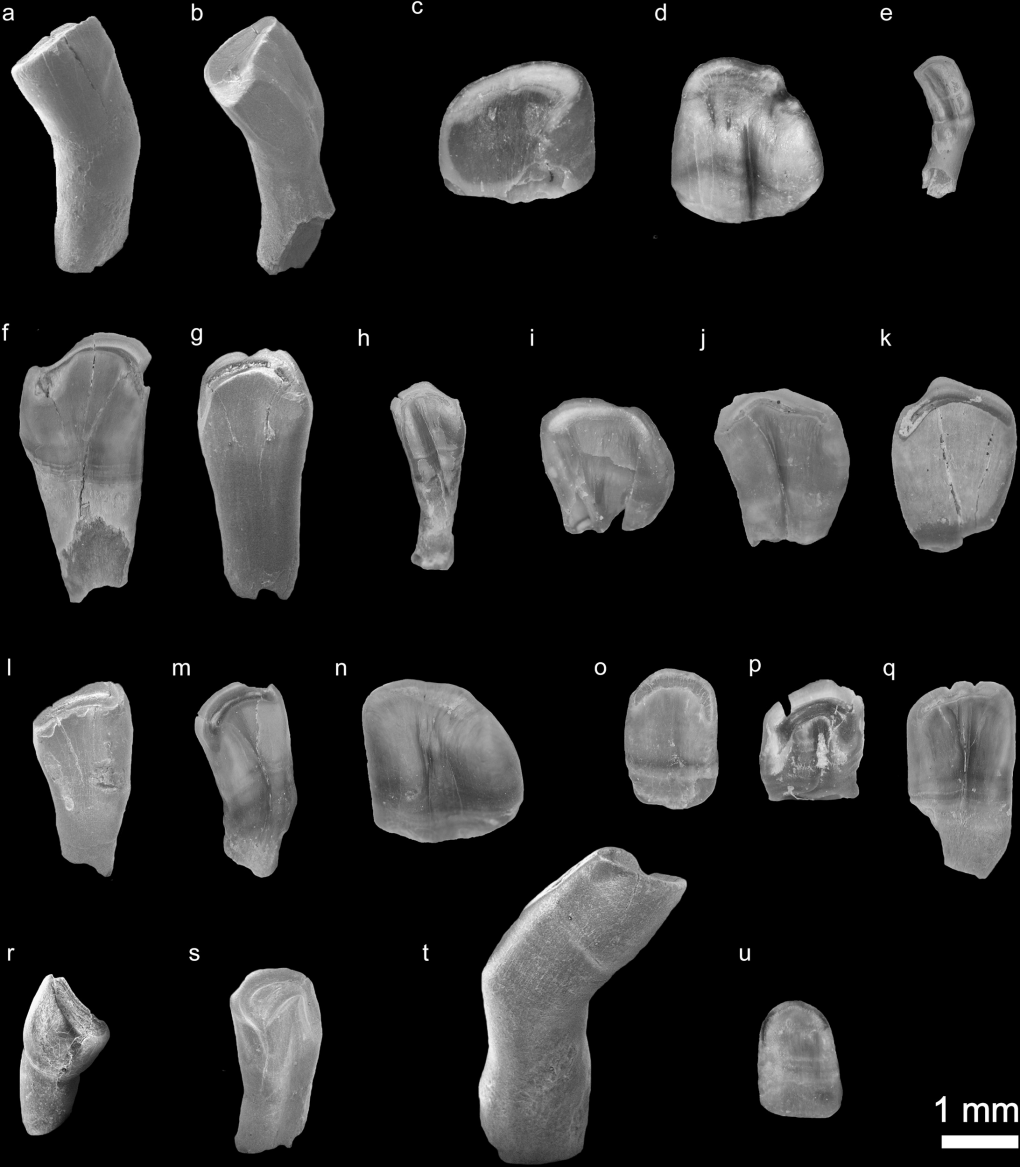
Isolated fossil pharyngeal teeth from the early Pliocene locality Ҫevırme (Erzurum Province, Tekman district). (**a-e**), species/clade diagnostic shape classes: **a,** shape class "A" characteristic of *C*. *umbla* (BGR Ҫevırme 1). (**b**), shape class "R", characteristic of *C*. *sieboldi* (BGR Ҫevırme 3). (**c-d**), shape class "J", characteristic of *C*. *baliki* (BGR Ҫevırme 4, 5). (**e**), clade diagnostic shape class "M", characteristic of Aralo-Caspian clade of genus *Capoeta* (*C*. *sevangi* and *C*. *capoeta*) BGR Ҫevırme 23). (**f-k**), genus diagnostic shape class "C" (BGR Ҫevırme 24, 25, 26, 27, 28, 29). (**l-s**), common shape classes shared by different species. (**l-n**), shape class "B" (BGR Ҫevırme 155, 156, 157). (**o-q**), shape class "F" (BGR Ҫevırme 195, 196, 197). (**r-s**), shape class "H" (BGR Ҫevırme 226, 227). (**t**), not identified, possibly tooth pathology (BGR Ҫevırme 237). (**u**), not identified (BGR Ҫevırme 238).

The studied fossil fish material is collected from 1965–1969 during the prospection of Neogene lignite deposits of Turkey [49]. The material contains 247 isolated pharyngeal teeth (Table 1, Fig 5A–5U) collected from the early Pliocene locality Ҫevırme in Eastern Anatolia, Turkey. From the same horizon, 41 isolated pharyngeal teeth of Leuciscinae (*Leuciscus* sp.) and some amphibian and reptile bones are also founded [28]. The studied fossil material already housed in the Bundesanstalt für Geowissenschaften und Rohstoffe, Hannover (BGR collection numbers) and no additional excavation of fossils was undertaken. The studied isolated fossil pharyngeal teeth are photographed under the Leica DVM5000 digital microscope, Leica M50 stereomicroscope and LEO Model 1450 VP scanning electron microscope (SEM). Recent comparative material (pharyngeal bones) is represented by adult individuals and comes from following collections: Bavarian State Collection for Anthropology and Palaeoanatomy Munich (SNSB), Palaeontological Collection of Tübingen University (GPIT), Senckenberg Naturmuseum Frankfurt (SMF) and National Museum of Natural Sciences of Madrid (MNCN) (Table 2). Both fossil and extant specimens publicly deposited in the above-mentioned collections and accessible by others in a permanent repository.

**Table 1:** Isolated fossil pharyngeal teeth (n = 247) and identified shape classes in the studied fossil material.

Specimens numbers	Shape classes	Tooth positions in tooth rows	Species	Depository
2	A	a2	*C*. *umbla*	BGR Ҫevırme 1 BGR Ҫevırme 2
40	B	a3, a4	*C*. *umbla*, *C*. *baliki*, *C*. *damascina*, *C*. *buhsei*	BGR Ҫevırme 155–194
131	C	a3-a5	all *Capoeta* species	BGR Ҫevırme 24–154
32	F	b1-b3, c1-c2	*C*. *sevangi*, *C*. *capoeta*, *C*. *sieboldii*, *C*.*trutta*, *C*. sp.	BGR Ҫevırme 195–226
9	H	b1, c1	*C*. *umbla*, *C*. *baliki*, *C*. *damascina*	BGR Ҫevırme 226–235
20	J	a2	*C*. *baliki*	BGR Ҫevırme 4–22
1	M	b1-b2, c1	*C*. *sevangi*, *C*. *capoeta*	BGR Ҫevırme 23
1	R	a2	*C*. *sieboldi*	BGR Ҫevırme 3
1	Not identified	a2	Not identified/ pathology	BGR Ҫevırme 237
10	Not identified	a2	Not identified (tooth genus)	BGR Ҫevırme 238–247

**Table 2:** Comparative material of extant *Capoeta* species.

Scientific name	Locality	Number of samples (n = )	Size (in cm)	Depository
*Capoeta sieboldii*	Kizilirmak River, town of Avanos, Turkey	1	40	GPIT-OS-00858
*Capoeta baliki*	Kizilirmak River, town of Avanos, Turkey	1	40	GPIT-OS-00859
*Capoeta trutta*	Assad Sea, Syria	2	40 40	SAPM-PI-02908, SNSB* SAPM-PI-02910, SNSB
*Capoeta capoeta*	Saghamo Lake, Georgia	13	23–25	GPIT-OS-00860*
*Capoeta umbla*	Khata River, Adyaman, eastern Turkey	1	36	SAPM-PI-00718, SNSB
*Capoeta sevangi*	Sevan Lake, Armenia	9	25–30	GPIT-OS-00861*
*Capoeta sp*.	Dokan Reservoir, Iraq	2	39,5 35	SAPM-PI-00719, SNSB* SAPM-PI-00721, SNSB
*Capoeta buhsei*	Soleghan River, Namak Lake, Tehran, Iran	1	15	AT241586, MNCN
*Capoeta saadii*	Shahpur River, Dalaki River, Bishapur, Iran	1	15	IR3, MNCN
*Capoeta damascina*	Homs or Qattinah Lake, Orontes River drainage, Syria	1	23	SYR08/25, SMF

*Collection numbers of scanned samples.

### Identification of isolated fossil pharyngeal teeth

**Fig 6:**
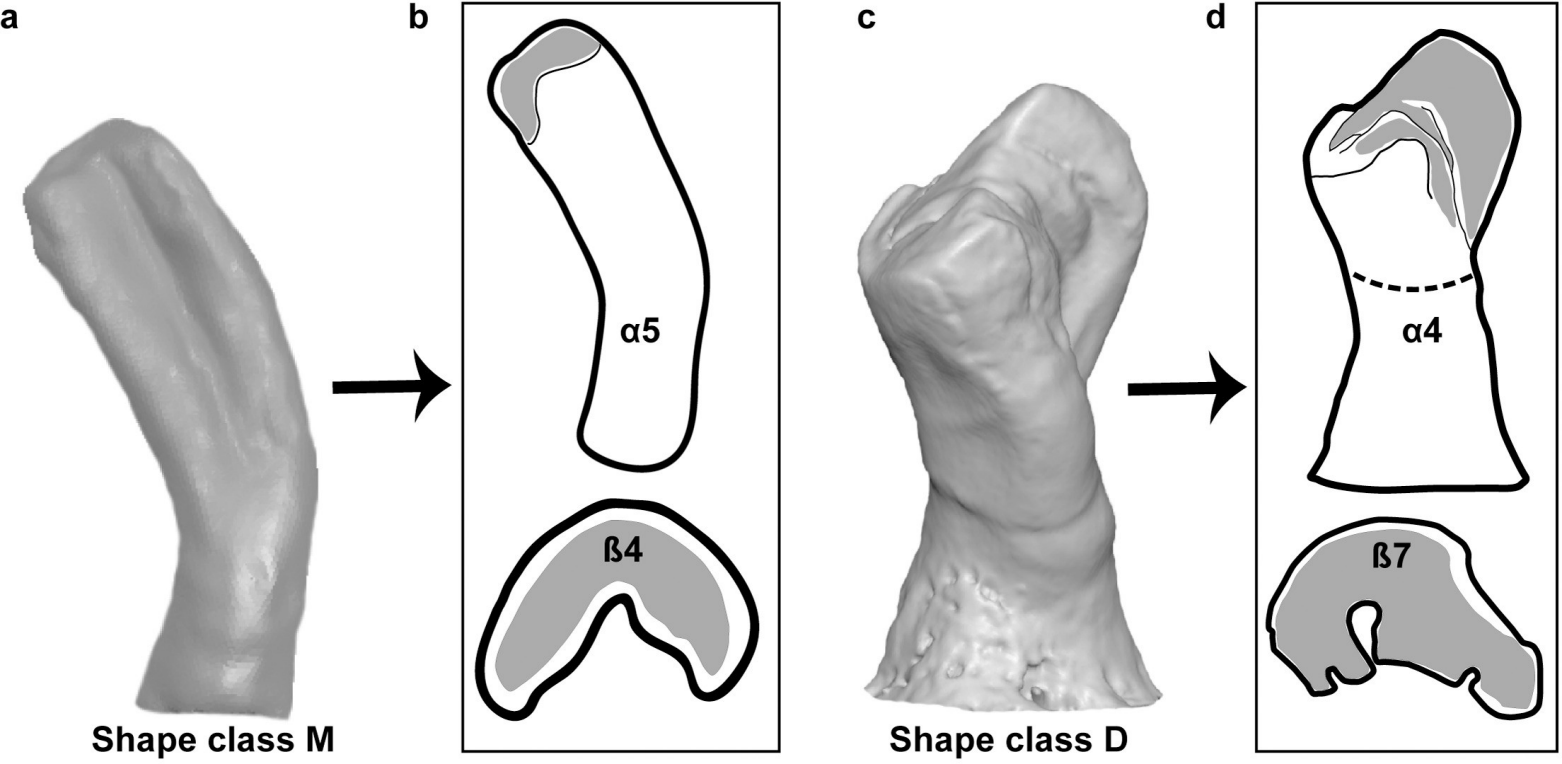
Examples to describe the isolated pharyngeal teeth based on the shape characters and shape classes. (**a**), shape class "M", b2 tooth of extant *C*. *capoeta*. (**b**), shape characters (α5β4) defining shape class "M". (**c**), shape class "D", a2 tooth of extant *C*. *sieboldi*. (**d**), shape characters (α4β7) defining shape class "D". Details of shape characters α and β see Ayvazyan et al. 2018. The scales are not given to avoid scaling up of the figures.

This study uses methodology and terminology established by Ayvazyan et al. (2018). The isolated fossil pharyngeal teeth are described and identified based on the established non-overlapping shape characters (αβ) and shape classes (A to R). Ayvazyan et al. (2018) applied an artificial (virtual) wear experiment in 3D software to check the possible effects of wear degree on the teeth morphology, its influence on the transverse cross section and other recorded characters (e.g. folded edge of grinding surface). The experiment shows that there is no any significant changes of transverse cross section during applied virtual wearing, while folds of the grinding surface can change during the wearing process: they deepen, enlarge, or disappear. Thus, these variable characters are not included in the description of the isolated fossil pharyngeal teeth. Examples of applied shape characters to identify shape classes are shown on Fig 6. The identified shape classes within the isolated fossil pharyngeal teeth are applied to identify the teeth according to the identification key (see S6 Fig in [9]).

### Nomenclature issues of isolated pharyngeal teeth of cyprinids

It is always an issue between molecular and morphologic studies regarding the nomenclature of taxa. This is further complicated if the material is composed by isolated pharyngeal teeth, thus restricting morphologic observations. To account for this uncertainty we prefer to use the open nomenclature system for fossil isolated cyprinid teeth.

### Ethics statement

No permits were required for the described study, which complied with all relevant regulations.

## Results

Systematic palaeontology

Clade Teleostei Müller 1846

Family Cyprinidae Rafinesque, 1810

Subfamily Barbinae Bleeker, 1859

Genus *Capoeta* Valenciennes, 1842

Material: 247 isolated fossil pharyngeal teeth collected from the Çevirme (Erzurum Province, Tekman district) locality (BGR Ҫevırme 1–247).

### Description

The studied isolated fossil pharyngeal teeth contain different tooth morphologies. They include eight main shape classes: "A", "B", "C", "F", "J" "H", "M" and "R" (details about shape classes see Ayvazyan et al. 2018).

Shape class "C" (Table 1, Fig 5F–5K) (n = 131)–the teeth are spatulate, the tooth body sharply widens distally. The foot-crown border is not well distinguished, and the foot is nearly two times narrower than the tooth crown. The teeth are characterized by a relatively narrow grinding surface, which reminds a hook. Some samples within the isolated fossil pharyngeal teeth can be identified as replacement teeth based on the presence of resorption traces (Fig 5I–5K).

Shape class "B" (Table 1, Fig 5L–5N) (n = 40)–the tooth body is spatulate, it is widens distally and the foot is narrower than the crown. The foot-crown border is not well distinguished. The grinding surface is comma-shaped and wider than the ones among the shape class "C". Besides this, these teeth have a deep groove on the grinding surface.

Shape class "F" (Table 1, Fig 5O–5Q) (n = 32)–these teeth are also spatulate in shape, the foot-crown border is not well distinguished. The grinding surface is reniform. In some specimens the edge of the grinding surface bears some hooks. Within this shape class, also replacement teeth are recorded (Fig 5P).

Shape class "J" (Table 1, Fig 5C and 5D) (n = 20)–is represented only by replacement teeth, despite this, the teeth morphology is clearly described by the shape characters α11 and β9. The crown is short, robust and convex posteriorly. A well-developed fold is present at the anterior part of the crown, which slightly divides it into two unequal parts. The grinding surface is ovate, but broad posteriorly.

Shape class "H" (Table 1, Fig 5R and 5S) (n = 9)–the teeth are molariform. The tooth body is compressed at the foot-crown border where it bends anteriorly. Due to this, the foot-crown border is well distinguished. The foot is longer than the crown but has nearly the same width as the crown. The crown is convex posteriorly. The grinding surface is triangular and possesses a visible groove.

Shape class "A" (Table 1, Fig 5A) (n = 2)–the tooth is oblong and widens slightly distally. The tooth body bends anteriorly at the foot-crown border. The tooth body is compressed and the foot-crown border well distinguished. The crown is slightly convex posteriorly. The grinding surface is triangular in shape with well visible groove on it.

Shape class "M" (Table 1, Fig 5E) (n = 1)–the tooth body is linear, the foot is shorter than the crown, but they have the same width along the tooth body. The tooth body bends laterally at the foot-crown border. The grinding surface is narrow and reniform.

Shape class "R" (Table 1, Fig 5B) (n = 1)–the tooth is molariform. The tooth body is compressed at the foot-crown border where it bends posteriorly. The foot and crown have nearly the same length, but the crown is wider than the foot. The grinding surface is reniform and possesses a deep groove.

An isolated fossil pharyngeal tooth (Table 1, Fig 5T) (n = 1) is not possible to attribute to one of delineated shape classes. We described this tooth as pathologic as the tooth crown bends extremely anteriorly, and we assume that this tooth possibly cannot participate in food processing.

The other recorded shape class (Table 1, Fig 5U) (n = 10) is represented only by replacement teeth and not found within recoded shape classes according to Ayvazyan et al. 2018. We marked these teeth as not identified. The crown is convex posteriorly and concave anteriorly. The grinding surface is half-moon in shape. These teeth are replacement teeth, based on their morphology we can assume, that they belong to the first teeth row and tooth position a2.

As it is shown in our previous study [9], tooth shape classes can be used for the identification of isolated pharyngeal teeth at generic and specific levels. Within studied fossil material, both genus and species diagnostic shape classes are present.

The shape class "C" (tooth position a3-a5) is identified by Ayvazyan et al. 2018 as the only genus diagnostic shape class for the genus *Capoeta*. Three shape classes in the studied samples (“A”, “J”, “R”) are considered species diagnostic. These shape classes occur in the first tooth row at tooth position a2 in extant *C*. *umbla*, *C*. *baliki* and *C*. *sieboldi* respectively. Four other recorded shape classes "B", "F", "H" and “M” occur in more than one species. Shape class "M" is clade diagnostic for both species of the Aralo-Caspian clade *C*. *capoeta* and *C*. *sevangi*. Finally, shape classes "B" and "H" are diagnostic for the four species of the Anatolian-Iranian clade or *damascina* complex (*C*. *saadii*, *C*. *buhsei*, *C*. *damascina*, *C*. *umbla* and *C*. *baliki*) [9]. The shape class “F” is characteristic of *C*. *trutta*, *C*. *sieboldi*, *C*. *capoeta* and *C*. *sevangi*.

### Percental distribution of the shape classes of the pharyngeal teeth

The distribution of the shape classes at the pharyngeal bone confines to a certain topology (S5 Fig in [9]). In order to be able to compare the studied fossils with recent teeth (overall 84 teeth from ten *Capoeta* species) and/or to estimate the number of potential species present in the material, the percental distribution of the shape classes within ten recent *Capoeta* species is calculated and compared with those in the fossil material [9].

In the recent species, the species diagnostic shape classes "A", "J" and "R", mainly occur in the first tooth row at the a2 position (except *C*. *sieboldi* which has also diagnostic shape class at the tooth position b1 [which is also included into percental estimation]), are rare and represent 3% out of 84 pharyngeal teeth of the ten recent species. The genus diagnostic shape class "C" (tooth positions a3-a5) builds 27% within the studied 84 pharyngeal teeth. The shape classes "C" and "B" (at the tooth positions a3-a5) make nearly 33% (S4 Fig in [9]).

In the fossil material, the shape class "C" can be referred to around 53% of all teeth; the shape classes "C" and "B" (a3-a5 positions) make 69%, whereas the species diagnostic shape classes "A", "J" and "R" (a2 tooth position) comprise 10% of studied isolated fossil pharyngeal teeth (S1 and S2 Figs).

**Fig 7:**
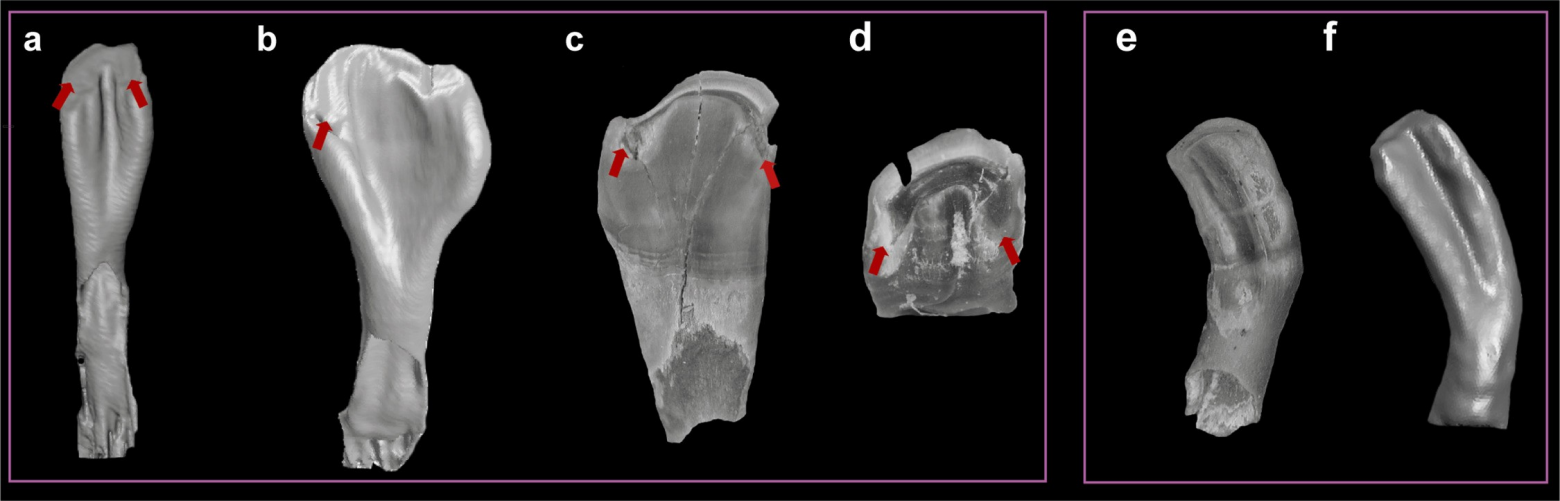
Additional morphological characters (besides the shape characters (αβ) in fossil and extant pharyngeal teeth (not to scale). (**a**), *Capoeta* sp., b3 tooth (extant) (SAPM-PI-00719, SNSB). (**b**), *C*. *trutta*, a5 tooth (extant) (SAPM-PI-02908, SNSB). (**c-d**), isolated fossil pharyngeal teeth (identified as shape class "C" and "F" respectively) (BGR 6, 16). (**e**), isolated fossil pharyngeal tooth (BGR 5). (**f**), *C*. *capoeta*, b2 tooth (extant) (GPIT-OS-00860^a^), both are identified as shape class "M". The ruptures of grinding surface are marked by red arrows (a, b, c, d) and an example of very similar tooth morphology in fossil (e) and extant (f) isolated pharyngeal teeth.

Morphological observations of isolated fossil pharyngeal teeth revealed, besides the main distinguished characters (lateral outline (α) and transverse cross-section (β)), further charcters commonly occuring within both recent and fossil *Capoeta*. They are "ruptures" of the grinding surface and the crenated edge of the grinding surface, which are variable and depending on the degree of tooth wearing (Fig 7) (details see Ayvazyan et al., 2018). These structures are not considered as a species characteristic.

## Discussion

In our fossil samples, we record eight shape classes where the genus diagnostic shape class “C” dominates the assemblage (53%). Identified shape classes as species or clade diagnostic (A, J, R, M) compose 10% of the assemblage (S1 Fig).

### Possible influence of plasticity and allometry on high diversity of recorded shape classes

The literature provides examples of the potential effects of plasticity on the dentary bone and tooth morphology mainly in cichlid fish cultures by applying contrasting diets (soft and hard) [50–53]. These studies recorded some degree of phenotypic plasticity of dentary bone morphology and in some cases tooth size. The influence of these two diets on the development of the cyprinid pharyngeal dentition is also tested in the benthophagous cyprinid black carp. Dietary did not change the tooth morphology, but, instead, it has been found that broad diet may influence the frequency of tooth replacement and size patterns [54]. These studies are mainly based on aquarium experiments in benthophagous species where two extreme diets (commercial fish as a soft and snails as hard food) are tested. Under natural conditions, fishes are not forced to feed on only one type of food. Thus, it is data can be applied to, in the present paper studied algae-scrapping species *Capoeta*, which are recorded from single geological layer and are sympatric individuals in a uniform environment. Considering this, the effect of feeding on different food should not be considered biasing on the carp pharyngeal tooth morphology, and, thus, we exclude the effect of plasticity on the studied fossil material.

Allometric shifts in pharyngeal tooth morphology cannot explain the high diversity of recorded shape classes in the studied fossil samples. Morphological shape remodeling in cyprinids happens in very early stages of their ontogeny. Juveniles (standard size of a few mm) have different tooth morphology than the adult samples, but the significant morphological changes are finalized in this early stage. Thus, the adult dentition in cyprinid fishes is completed by at the later larvae or juvenile stages [55]. Our fossil material is represented by adult individulas, as the studied fossil pharyngeal teeth sizes vary between 0.8–3 mm (it is a sampling artifact introduced by mesh size limitation washing collection technique). Therefore, our fossil samples is composed of isolated pharyngeal teeth of adult individuals.

### Taxonomic assignment

For species-level taxonomy we discuss two possible interpretations. The assemblage can be interpreted to document either a single, very heterodont species or several *Capoeta* species.

#### 1. The fossil assemblage documents one species

The recent *Capoeta* species are characterized by different degree of heterodonty, which varies between three and six shape classes per species. For instance, *C*. *damascina*, the most heterodont extant species, is characterized by six different shape classes [9]. The second most heterodont species *C*. *umbla* (Heckel, 1843) is characterized by five different shape classes, four of them are shared with *C*. *damascina*. Eight shape classes, as found in our fossil samples, is unprecedented among extant species. It is also highly unlikely that a fossil species shows this degree of heterodonty, given the ten tooth positions at pharyngeal bones are present. Therefore, we consider the ‘single species’ interpretation as rather unlikely.

#### 2. The fossil assemblage represents more than one species

The specific identification of extant *Capoeta* species is possible only on the morphology of the teeth at the tooth position a2 [9]. The Çevırme association contains four shape classes, which are species-specific among recent taxa at the a2 position: the shape class “A” characterizes *C*. *umbla*, the shape class “J” is typical for *C*. *baliki* (both species belong to the Anatolian-Iranian clade) and the shape class “R” is found only in *C*. *sieboldi* (*sieboldi* clade). The shape class “M” is shared at the a2 position by two closely related Aralo-Caspian species *C*. *capoeta* and *C*. *sevangi*. Therefore, we assume that the Çevırme assemblage is constituted of four species.

The four discussed extant species are also characterized by other shape classes, which are not found within the studied fossil material. The shape class “I” is common in *C*. *umbla* and *C*. *baliki*, it occurs at the topological positions b2, b3 and c2. These teeth are small and may not be found due to taphonomic or sampling bias (tooth diameter is smaller than 0.8 mm). Two additional shape classes “N” and “O”, which are missing in our sample, characterize the two Aralo-Caspian *Capoeta* species *Capoeta sevangi* and *Capoeta capoeta*, at the tooth position a2. We interpret the lack of these species characteristic shape classes by younger divergence of these species (see below).

Our results indicate the presence of possible four species in the fossil assemblage, which belong to three different clades (Anatolian-Iranian, Aralo-Caspian, and *sieboldi* clades) of the genus *Capoeta*. According to all molecular studies [29, 56, 57], these three clades are monophyletic and sister groups to the Mesopotamian clade (Fig 3a).

### The evolution of the genus *Capoeta* as a species flock scenario

Greenwood (1984) suggests that, in order to identify a group of organisms as species flock, the representatives should be monophyletic and endemic to an area they inhabiting [21]. Later on, five main criteria are distinguished to detect the flock species [13, 58]: 1) monophyly, 2) high species diversity (speciosity), 3) high level of endemism, 4) morphological and ecological diversity; and 5) habitat dominance in terms of biomass. A later study [59], suggests to concentrate on three robust, easier to determine criteria such as monophyly, endemism and speciosity. This study suggests ranking the ecological criterion as secondary. Our fossil *Capoeta* samples correspond to all five criteria sensu Eastman and McCune (2000) and can thus be regarded as a species flock. The extant *Capoeta* is a monophyletic phytophagous barbin genus, widely distributed in West Asian and the Ponto-Caspian water basins and comprise 30 extant species [5, 29, 35, 56]. Our four fossil species (*Capoeta* cf. *umbla*, *C*. cf. *baliki*, *C*. cf. *sieboldi*, *C*. sp. *capoeta*/*sevangi*) belong to a monophyletic clade composed of *Capoeta sieboldi*, Anatolian-Iranian and Aralo-Caspian species (Fig 3A) endemic to the drainage systems of the Black and Caspian seas and Persian Gulf (Fig 2), thus, fulfilling the three main criteria for species flock recognition [59]. Certainly, we cannot be fully definite that our fossil taxa are also monophyletic. However, considering that the phylogenetic analysis using the morphology of extant pharyngeal teeth [9] placed the species in the same topology as the molecular phylogenetic analysis, we are confident that the fossil species attribution correspond to extant taxa. Nevertheless, as in every biological study species identification retain certain degree of uncertainty, which would potentially affect the probable monophyly of the fossil taxa.

The endemic occurrence of the genus *Capoeta* in Western Asia and the Ponto-Caspian region is supported by its exclusive extant and fossil record in the region [7, 30, 60–62]. The taxonomic studies of this genus show the morphological and meristic diversity of the extant *Capoeta* species [9, 39, 63–65], but detailed ecologic studies are lacking so far. The fifth criteria (habitat dominance in terms of biomass) is more difficult to access for the fossil palaeocommunity. However, within the studied samples from the locality Ҫevırme *Capoeta* dominates not only by the species richness over *Leuciscus* (one undetermined medium-size species), but also in terms of numbers of specimens (247 *Capoeta* teeth versus 41 *Leuciscus* teeth), suggesting habitat dominance of *Capoeta* in the Tekman Palaeolake of the Işıklar Formation 4 Ma ago.

Our results are largely in agreement with estimated divergence times within *Capoeta* [57], showing that at 4 Ma *C*. *sieboldi* is already diverged and the Aralo-Caspian clade species *C*. *capoeta* and *C*. *sevangi* are not yet separated, which explains the lack of their species-specific tooth shape classes "N" and "O". The fossil Aralo-Caspian clade taxon may, therefore, represent a newundescribed species ancestral to the extant members of this clade. However, published divergence times seem to be overestimated since the fossil calibration points used for the molecular clock are too old, maybe by a factor of two *Barbus* sp. set at 18 Ma citing Böhme & Ilg 2003 refer in fact to *Barbus* s. l., which is probably closer related to *Cyprinion*; the oldest *Barbus* s. s. fossils are known from sediments of age at least 8 Ma, Böhme unpublished data) [66]. Nevertheless, the oldest unequivocal *Luciobarbus* with affinities to *L*. *subquincunciatus* (the sister clade of *Capoeta*, Fig 3A and 3B) is *L*. *vindobonensis* from 9.8 Ma old deposits in Austria [67], suggesting that the evolution of *Capoeta* is largely a late Miocene event.

The presence of a four-million-year old *Capoeta* species flock in the Tekman Basin with members of three recent clades is very remarkable. We hypothesize, that the Tekman Palaeolake, which was part of a large Armenian Highland lake system, was a place of the speciation of *Capoeta* species related to the three recent clades of the genus (Anatolian-Iranian, Aralo-Caspian and *sieboldi*). Moreover, the huge Armenian Highland lake system, which formed during the late Miocene and represents the source of all major rivers in Western Asia and the Ponto-Caspian region where *Capoeta* is widely distributed, could represent the centre of origin of *Capoeta* including its Mesopotamian clade.

A recent study shows that tectonic reorganization in the region, starting about the Miocene-Pliocene transition (ca. 5.5 Ma) along the East and North Anatolian faults [46, 68]. It resulted in substantial surface uplift and probably caused the gradual reshaping of the hydrological network in the area. This could largely contribute to dispersal and further speciation of the members of the species flock into their distribution areas nowadays.

**Fig 8:**
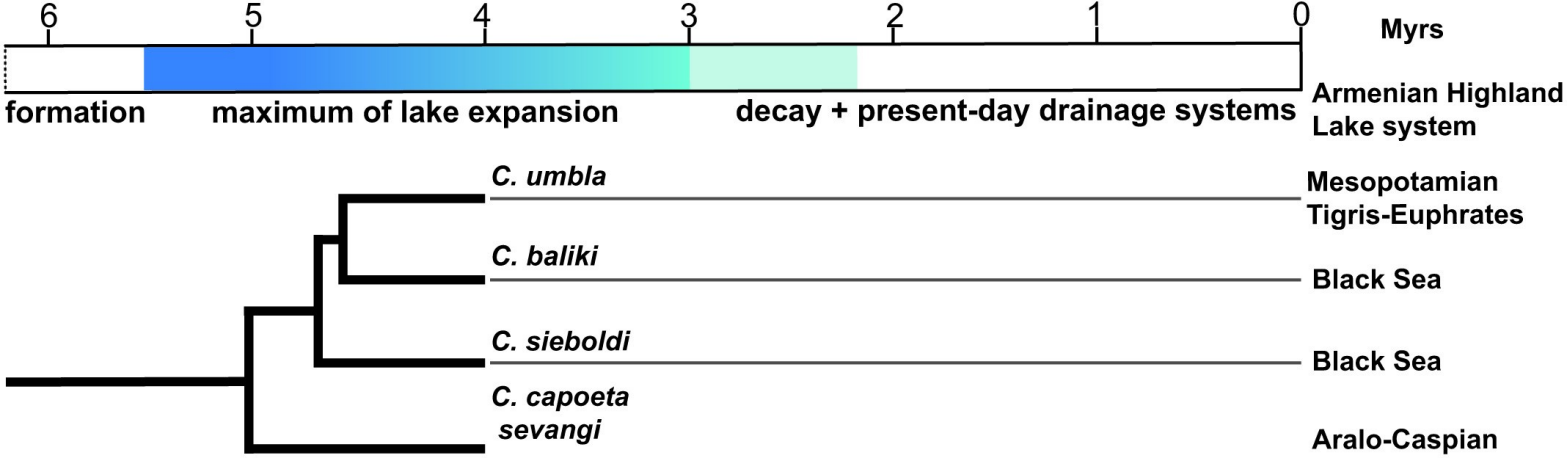
Hypothetical evolutionary stages of the palaeolake system of Armenian Highland since latest Miocene. Three main stages are suggested (marked by blueish colours): formation, maximum of lake expansion, decay and fully development of present-day drainage system. The monophyletic clade of recorded species within the fossil material shows the presence of the species flock of *Capoeta* at 4 Ma ago in palaeolake system of Armenian Highland.

The possible species flock scenario of the genus *Capoeta* as well as the reorganization of the palaeolake system in Armenian Highland are hypothetically illustrated in Fig 8, where three main stages of lake evolution.

The other possible explanation of our results could be the concept of secondary contact. This scenario (speciation of hybrids) is very similar to the above suggested species flock model, however, without any genetic information we cannot be precise about this hypothesis. More studies and more fossil sites inside and outside distribution area of *Capoeta* are needed to test our hypothesis, but according to the current available data, the fossil species flock interpretation is the most plausible.

## Conclusions

For the first time, a detailed study of the isolated fossil pharyngeal teeth of the genus *Capoeta* (n = 247) is provided. The description and identification of the fossil material from Ҫevırme (Erzurum Province, Tekman district) is based on the methodology introduced by Ayvazyan et al. 2018. We show that our methodology is applicable to the fossil record of the genus *Capoeta* and allows identification of the isolated fossil pharyngeal teeth at species level. Within the studied fossil material eight shape classes are distinguished, four of them are species or clade diagnostic and indicate the presence of the four sympatric *Capoeta* species (*C*. cf. *sieboldi*, *C*. cf. *umbla*, *C*. cf. *baliki* and *C*. sp. *capoeta*/*sevangi*) in the Tekman Palaeolake at 4 Ma. These four species belong to a monophyletic clade of the genus and today they are distributed in different water basins (Euphrates/Kura/Black Sea) of Western and Ponto-Caspian region. We interpret this high local diversity of closely related species in terms of the species-flock model.

Literature review suggests that the Tekman Palaeolake was part of an unrecognized huge late Miocene to Pliocene palaeolake system in the present-day Armenian Highland and we hypothesized that the evolution of *Capoeta* occurred there during the late Miocene. Pliocene tectonic activities disrupted this lake system and resulted in the very characteristic biogeographic distribution of *Capoeta* in West Asian and Ponto-Caspian drainage systems today.

## Supporting information

S1 Fig Frequency distribution of recorded shape classes in the Ҫevırme sample (n = 247). (TIF)

S2 Fig The frequency (in % of all studied teeth, n = 247) of the tooth positions for isolated fossil pharyngeal teeth from Ҫevırme. (TIF)

S1 Table The distribution of the extant ten *Capoeta* species, used for comparison. (PDF)
