# Supplementary figures and images for: Possible species-flock scenario for the evolution of the cyprinid genus Capoeta (Cypriniformes: Cyprinidae) within late Neogene lake systems of the Armenian Highland
Source: PLoS One. 2019 May 8;14(5):e0215543. doi: 10.1371/journal.pone.0215543 (PMC6505951; doi:10.1371/journal.pone.0215543)

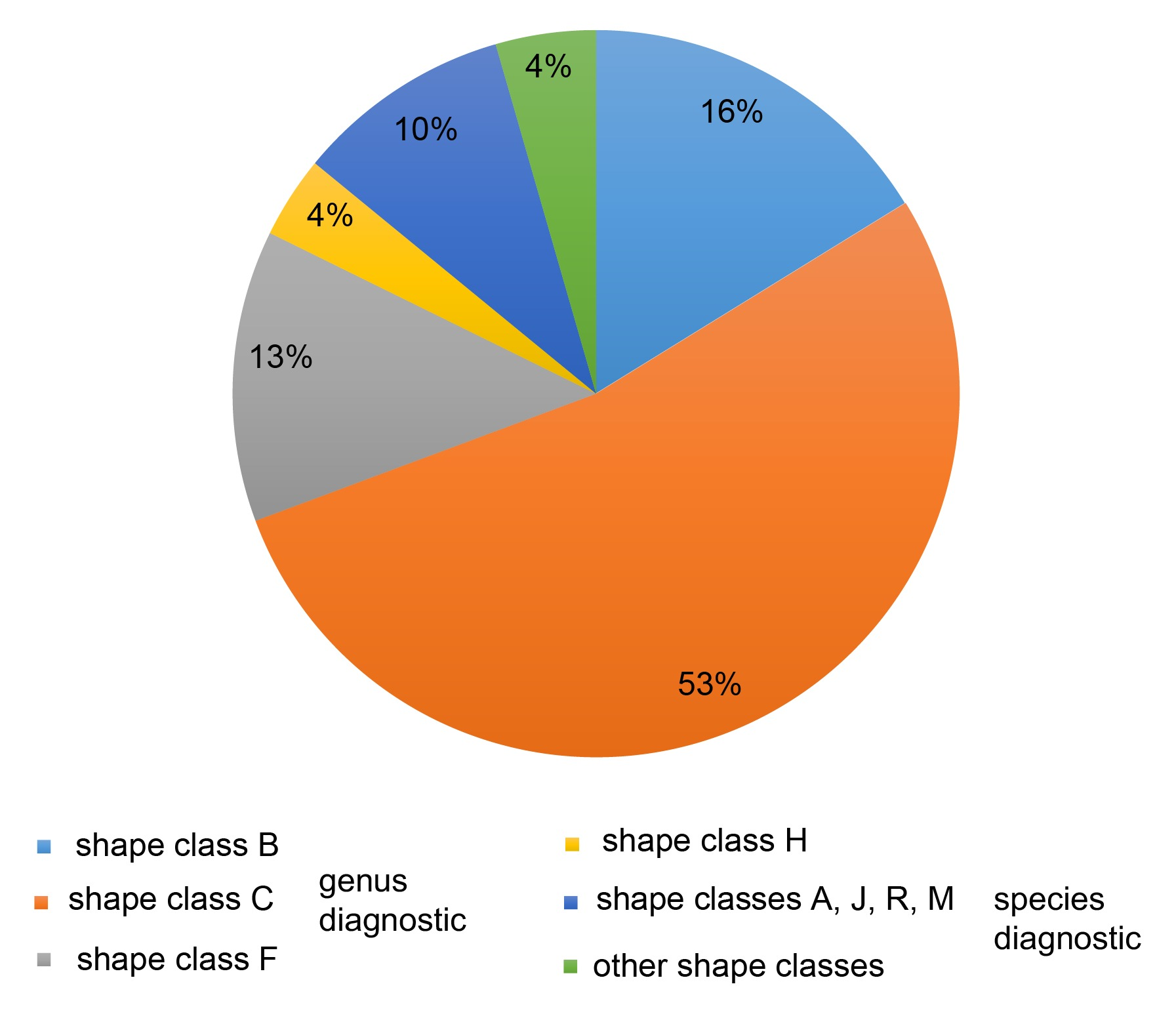

Supplement: S1 Fig — (TIF) [file pone.0215543.s001.tif]

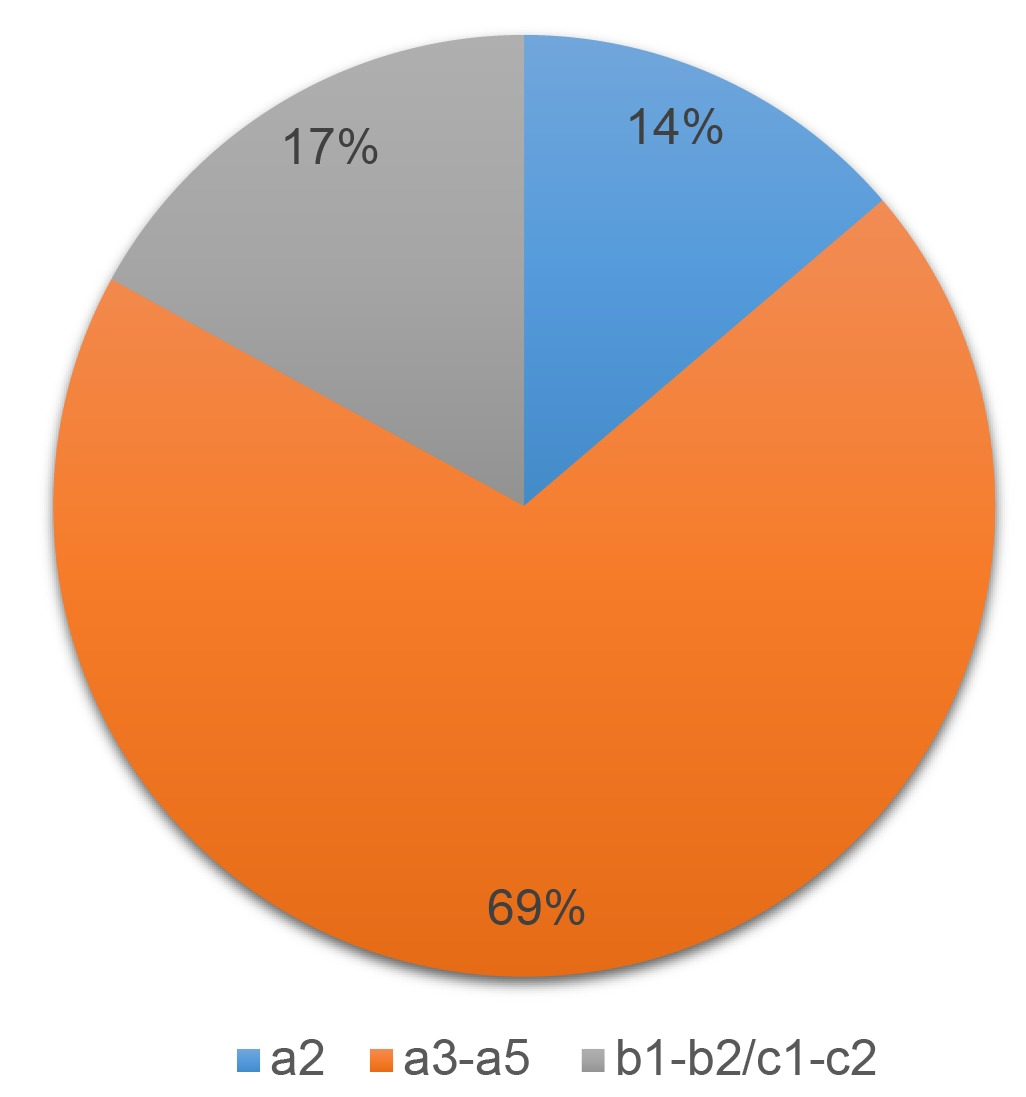

Supplement: S2 Fig — (TIF) [file pone.0215543.s002.tif]
