# Supplementary material for: Possible species-flock scenario for the evolution of the cyprinid genus Capoeta (Cypriniformes: Cyprinidae) within late Neogene lake systems of the Armenian Highland
Source: PLoS One. 2019 May 8;14(5):e0215543. doi: 10.1371/journal.pone.0215543 (PMC6505951; doi:10.1371/journal.pone.0215543)
Supplement: S1 Table — (PDF) [file pone.0215543.s003.pdf]

S1 Table. The distribution of the extant ten *Capoeta* species, used for comparison.

Species	Rivers/Lake	Water basins	Country	Source
C. umbla	Tigris, Euphrates	Persian Gulf	Iran, Iraq, Syria, Turkey	Freyhof J., 2014<br>Esmaeili H. R. et al., 2016
C. saadii	in almost all Iranian basins except of Sistan and Mashkid		Iran	Alwan N.H. et al., 2016
C. buhsei	Namak		Iran	Coad, B.W., 1998
C. baliki	Sakarya and Kızılırmak		Turkey	Turan D. et al., 2006
C. trutta	Tigris, Euphrates		Iran, Iraq, Syria, Turkey	Coad B.W., 1996
C. damascina	Jordan River drainage basin, the entire water bodies of Levant, Mesopotamia and partially southern Turkey		Iran, Iraq, Syria, Turkey, Jordan, Israel	Krupp F. and Schneider W., 1989
C. sieboldi	Kızılırmak	Black Sea	Turkey	Berg L.S., 1964
C. capoeta	Kura and Arax drainages, Lake Sevan and Orumiyeh basin	Caspian Sea	Afghanistan, Armenia, Azerbaijan, Georgia, Iran, Pakistan, Turkey, Turkmenistan	Talwar P.K. and Jhingran A.G., 1991
C. sevangi	Sevan Lake		Armenia	Zareian H. et al., 2016
